# Evaluation of the efficacy of trigger points combined with extracorporeal shock waves in the treatment of plantar fasciitis: heel temperature and plantar pressure

**DOI:** 10.1186/s12891-024-07296-2

**Published:** 2024-03-02

**Authors:** Bo Wang, Xiao-Lei Wang, Yan-Tao Ma, Wei Wu, Yong-Jun Zheng

**Affiliations:** 1 Department of Pain Management, Huadong Hospital affiliated to Fudan University, 221 West Yan’an RD, Shanghai, China; 2 Department of Elite Sport, School of Athletic Performance, Shanghai University of Sport, 188 Hengren RD, Shanghai, China

**Keywords:** Plantar fasciitis, Infrared thermal imaging, Shock wave, Plantar pressure

## Abstract

**Background:**

Plantar fasciitis (PF) is the most common cause of heel pain. Among conservative treatments, extracorporeal shock wave therapy (ESWT) is considered effective for refractory PF. Studies have shown that applying ESWT to the trigger points (TrPs) in the triceps surae may play an important role in pain treatment in patients with PF. Therefore, the purpose of this study was to combine the concept of trigger points and ESWT to explore the effect of this combination on plantar temperature and pressure in patients with PF.

**Methods:**

After applying inclusion and exclusion criteria, 86 patients with PF were recruited from the pain clinic of Huadong Hospital, Fudan University and randomly divided into experimental (*n* = 43) and control groups (*n* = 43). The experimental group was treated with extracorporeal shock waves to treat the medial heel pain point and the gastrocnemius and soleus TrPs. The control group was only treated with extracorporeal shock waves at the medial heel pain point. The two groups were treated twice with an interval of 1 week. Primary measurements included a numerical rating scale (NRS) score (overall, first step, heel pain during daily activities), and secondary measurements included heel temperature, Roles–Maudsley score (RMS), and plantar pressure. All assessments were performed before treatment (i.e., baseline) and 6 and 12 weeks after treatment.

**Results:**

During the trial, 3 patients in the experimental group withdrew from the study, 2 due to interruption of the course of treatment by the COVID-19 epidemic and 1 due to personal reasons. In the control group, 3 patients fell and were removed due to swelling of the heel. Therefore, only 80 patients with PF were finally included. After treatment, the two groups showed good results in NRS score (overall, first step, heel pain during daily activities), RMS, and plantar temperature, especially in the experimental group, who showed a significantly better effect than the control group.

**Conclusion:**

ESWT of the heel combined with the triceps trigger point of the calf can more effectively improve the pain, function and quality of life of refractory PF than ESWT of the heel alone. In addition, ESWT of the heel combined with the triceps trigger point of the calf can effectively reduce the skin temperature of the heel on the symptomatic side, indicating that the heel temperature as measured by infrared thermal imaging may be used as an independent tool to evaluate the therapeutic effect for patients with chronic PF. Although extracorporeal shock waves combined with TrPs treatment can cause changes in the patients’ gait structure, plantar pressure is still difficult to use as an independent tool to evaluate the therapeutic effect for PF.

**Trial registration:**

Registered in the Chinese Clinical Trial Registry (www.chictr.org.cn) on 12/17/2021 with the following code: ChiCTR-INR-2,100,054,439.

**Supplementary Information:**

The online version contains supplementary material available at 10.1186/s12891-024-07296-2.

## Background

Plantar fasciitis (PF) is a degeneration of the plantar fascia resulting from repetitive microtears that lead to an inflammatory reaction and not a primary inflammatory process as most believe it to be [1]. In most cases, PF is a self-limiting disease, but complete remission of symptoms may take up to 1 year [2].

PF can be treated by surgery or conservative treatment [3]. Due to the large trauma associated with surgical treatment and the risk of postoperative complications, conservative treatment is the first choice in clinical practice [4]. Conservative treatment is effective for approximately 90% of patients [5] and includes nonsteroidal anti-inflammatory drugs, plantar corrective insoles, physical therapy, stretching, corticosteroid injections, etc. Extracorporeal shock wave therapy (ESWT) is considered the main means of conservative treatment for PF, providing substantial symptom relief for most patients [6]. However, most ESWT schemes commonly used to treat PF only focus on the plantar fascia and do not consider the tension of the entire lower limb, and the curative effect is often of a short duration.

At present, a relationship between trigger points (TrPs) and PF symptoms is acknowledge [7–9]. TrPs are sensitive nodules in the muscle or fascia that are painful when palpated, resulting in distal referred pain and autonomic nerve response [8, 10]. TrPs are mainly formed by excessive use of skeletal muscle and can be divided into two states: activated or recessive. Activated TrPs cause local area pain and referred pain, while recessive TrPs require mechanical stimulation to cause pain [8, 11]. At present, there are few studies on the use of extracorporeal shock waves combined with TrPs in the treatment of PF. Commonly used evaluation methods, such as the digital scoring method, are subjective and cannot objectively reflect the therapeutic effect [12, 13].

Some studies have suggested that PF may be caused by inflammation [14], whose reduction or aggravation can be reflected by a decrease or increase in temperature, respectively. The presence of TrPs induces excessive excitation of the sympathetic nerve, whose main function is to regulate blood vessels, resulting in local skin temperature changes [15]. Therefore, when the distribution of blood vessels in the heel changes, the temperature of the heel will change accordingly. Other studies have suggested that PF may be caused by high tension stimulation of the plantar fascia overload, and the presence of TrPs will cause higher tension than that in normal tissue [12]. In this study, we hypothesized that ESWT combined with TrPs treatment would reduce the heel temperature and cause changes in plantar pressure parameters in patients with PF. Therefore, our aim was to investigate the effect of extracorporeal shock waves combined with TrPs on plantar temperature and pressure in patients with PF.

## Methods

### Study design

A single-blind trial with 12 weeks of follow-up for parallel groups was carried out from August 2021 to August 2022. The study was conducted according to the principles of the Helsinki Declaration and approved by the Ethics Committee of Huadong Hospital Affiliated with Fudan University: No. 2021K109. This randomized controlled trial (RCT) has been registered at the Primary Registry of International Clinical Trial Registry Platform World Health Organization “Chinese Clinical Trial Registry” [ChiCTR2100054439]. Before participating in the project, the purpose of the study was explained to all patients, and written informed consent was obtained from all patients.

### Sample size

Due to the lack of previous studies, this study estimated the sample size through a preexperiment. This study is an RCT of a parallel design, with a numerical rating scale (NRS) as the main observation index, which is a continuous variable. The sample size was calculated by PASS 15 software. The test level parameter was set to α = 0.05 (bilateral), and the test efficiency parameter was set to 1-β = 80%. The minimum sample size required for the experimental group and the control group was calculated as N1 = N2 = 23 cases. The exit rate was set to 20%, so at least 29 people should be included in each group.

### Participants

**Fig. 1:**
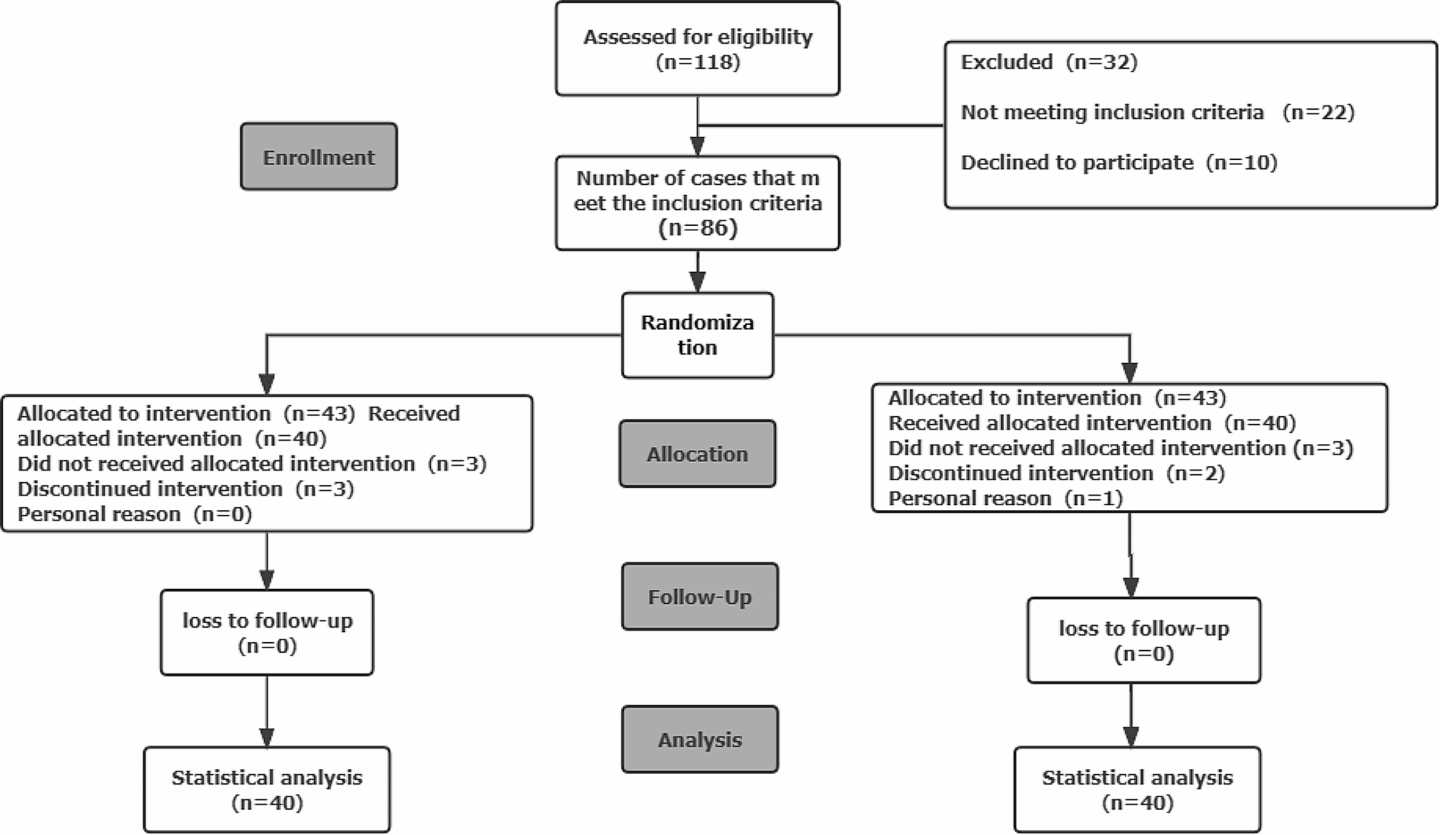
CONSORT flow chart for patient enrollment

A total of 118 participants were assessed for eligibility. Eighty-six patients with PF were recruited from the Department of Pain Management, Huadong Hospital Affiliated with Fudan University. All of the patients refused to undergo any surgery and signed informed consent forms. The CONSORT flow diagram for an RCT to evaluate the efficacy of extracorporeal shock waves combined with TrPs in patients with PF is illustrated in Fig. 1.

All patients voluntarily participated in the experiment. The inclusion criteria were as follows: (1) 18 years of age or older; (2) heel pain lasting ≥ 3 months; and (3) pain in the first step after waking up in the morning or obvious pain in the medial calcaneal tubercle and the starting point of the plantar fascia at 2–3 cm after standing for an extended period as well as B-mode ultrasonography showing plantar aponeurosis thickening greater than 4 cm and a low echogenic area [16] and an NRS score ≥ 5 points; (4) ineffective previous conservative treatment (nonsteroidal anti-inflammatory drugs and/or other analgesics, exercise programs, insoles); and (5) signed informed consent. The exclusion criteria were as follows: (1) previous history of ankle and foot fracture or surgery, foot and ankle infection, or history of lower limb tumor; (2) lower extremity neurological dysfunction; (3) rheumatic diseases and metabolic diseases; and (4) local injection of steroids or surgical treatment within three months.

### Randomization and blinding

Using a computer-generated random allocation sequence, 86 patients with PF were randomized and assigned into two equal groups by an independent researcher: an experimental group and a control group [17]. An independent researcher who was not involved in the recruitment, assessment, or intervention process conducted the randomization and was blinded to the group allocation. Participants were also instructed not to reveal their group assignment. Sequentially numbered sealed envelopes were used for allocation. The envelopes were opened only by the researcher responsible for applying the treatment programs. In group A, the experimental group, the treatment target was selected as the ipsilateral TrPs (gastrocnemius and soleus TrPs) and heel medial area pain points, while in group B, the control group, the treatment target was selected as the pain point in the medial area of the affected heel. Both groups were treated with a Swiss Storz radial shock wave device. The energy flux density is usually considered the main variable reflecting the physical and biological effects to maintain the consistency of treatment. A total of 3,000 shocks with a 15 mm treatment probe were selected. 8 Hz), and the treatment intensity was 1.4 bar.

### Outcome measures

Patients were measured and evaluated by a single person to reduce errors. All patients were assessed with the digital NRS, the Roles–Maudsley score (RMS), and infrared thermal imaging to measure heel temperature and plantar pressure before treatment (i.e., baseline) and 6 weeks after treatment and with the NRS, the RMS and infrared thermal imaging to measure heel temperature at 12 weeks after treatment.

### Primary outcome measures

The NRS score was used to evaluate overall heel pain, heel pain at the first step, and heel pain during daily activities in patients with PF. A score of 0 was considered no pain, 1–3 was considered mild pain, 4–6 was considered moderate pain, and 7–10 was considered severe pain.

### Secondary outcome measures

The RMS was used to assess the patients’ subjective satisfaction with the treatment. An RMS score of 1 point indicates excellent quality of life, an RMS score of 2 points indicates good quality of life, an RMS score of 3 points indicates acceptable quality of life, and an RMS score of 4 points indicates poor quality of life.

**Fig. 2:**
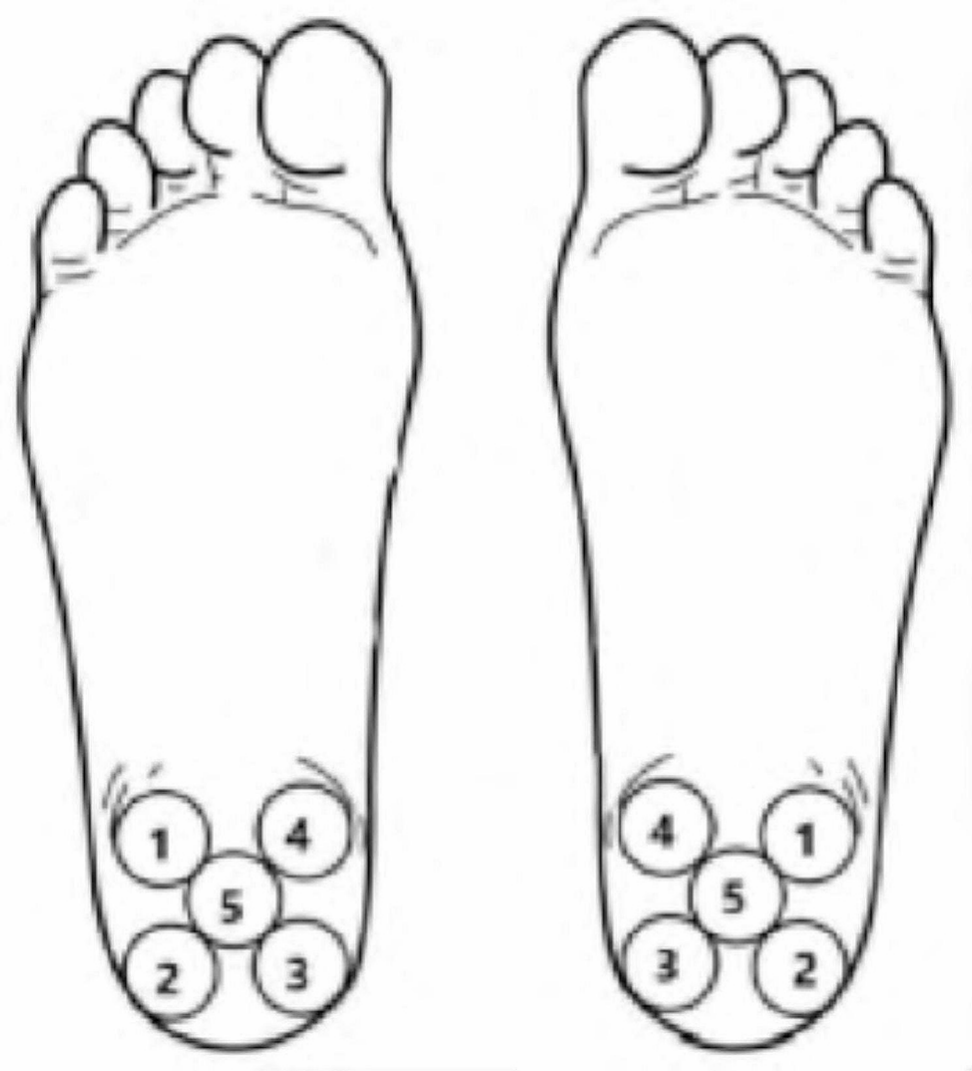
Five regions of interest in the heel region

The heel temperature was measured by infrared thermal imaging at 5 regions of interest according to the anatomical structure of the plantar surface (Fig. 2): outer/upper, position 1; outside/down, position 2; inside/above, position 3; inside/under, position 4; and central, position 5. The average temperatures of the five regions of interest were recorded as T1, T2, T3, T4 and T5, respectively.

Plantar forces and pressures were recorded during standing and level barefoot walking using a Sensor Medica plantar pressure gait analysis system (Sensor Medica Inc. Italy). This multistep barefoot analysis system used an 8 mm thick floor mat (map: 1600 × 400 mm) comprising 25,600 resistive sensors (four sensors per cm^2^) and samples data at a frequency of 400 Hz. The participants were told to stand barefoot on the plantar pressure test board, feet shoulder width apart, eyes facing forward, and hands on both sides while holding still for 30 s. When testing the plantar pressure during walking, each subject is required to first stand at a distance of 1.5 m from the plantar pressure plate and then walk barefoot in a straight line along the laid cushion. When walking, the patient is asked to maintain their usual speed and raise their head and chest until they reach the end of the cushion and return again. To increase the accuracy of the data, participants can walk 5 rounds on their own before the test starts, adapting to the surrounding environment before starting the test. The plantar pressure data in the static state and walking state were collected, including the average static plantar pressure (forefoot and hindfoot), the maximum static plantar pressure (forefoot and hindfoot) and the dynamic inner and outer plantar loads.

### Interventions

#### Experimental group

According to the internationally recognized three principles of trigger point positioning theory, the following were used for location: (1) palpable skeletal muscle on tight band nodules; (2) obvious tenderness points on the tight bandage; and (3) reappearance of the patient’s symptoms of pain during palpation. If a point satisfies the above three conditions, it is considered an active trigger point. If it satisfies both 1 and 2 but does not satisfy 3, it is considered an implicit trigger point [8]. The patient’s gastrocnemius muscle, soleus muscle activity and hidden TrPs were identified and marked with a medical sterile surgical marker pen. To evaluate the trigger point position at the next session, the patient was asked not to clean the marker mark. Diagnostic criteria were applied by physical therapists with five years of experience in myofascial pain. The Swiss Storz-MP200 radial ESWT device was used once a week for a total of 2 treatments. The TrPs of the triceps surae and the pain points in the medial area of the heel were taken as the treatment points. Each patient was placed in a prone position, and the patient’s hands were placed on the side of the body to fully expose the affected leg and heel. The coupling agent was applied at the specified position, the leg was subjected to vertical impact, the heel was subjected to lateral impact, and 300 impacts were applied at each trigger point.

#### Control group

The subjects were treated with the Swiss Storz-MP200 radial ESWT at the medial heel pain point identified after palpation; the course of treatment, frequency and dose were the same as those of the experimental group.

### Statistical analysis

Statistical Package for Social Science 23.0 was used for statistical analysis. Continuous variables that conformed to a normal distribution are expressed as the mean and standard deviation. The Kolmogorov–Smirnov (K-S) test was used to test whether the data obeyed a normal distribution. If the data obeyed a normal distribution, the independent t test was used to compare data between groups; otherwise, a nonparametric test was used, and the Mann‒Whitney U test was used for pairwise comparisons. Categorical variables (sex) are expressed as frequencies and composition ratios, and the chi-square test was used to compare groups. Repeated measurement analyses were implemented for efficacy indicators that met a normal distribution and homogeneity of variance. If the Mauchly sphericity test was successful, two-factor analysis of variance was used; otherwise, the Greenhouse‒Geisser method was used for result correction. In the results of repeated measures analysis of variance, if there was no interaction effect between time and treatment factors, the main effect test was directly used to To evaluate the trigger point position evaluate the effect of treatment factors. If there was an interaction effect between time and treatment factors, the individual effect was analyzed; that is, the intragroup effect was analyzed by one-way repeated measurement analysis of variance, and the intergroup effect was analyzed by multivariate analysis of variance. *P* < 0.05 indicated that the difference was statistically significant, and *P* < 0.01 indicated a significant difference.

## Result

### Baseline characteristics

A total of 86 patients were included in this study. Among them, 3 patients in the experimental group withdrew from the study, 2 due to the interruption of the course of treatment affected by COVID-19 epidemic and 1 due to personal reasons. In the control group, 3 patients fell and were removed due to swelling of the heel. Finally, a total of 80 patients completed the trial. There were 30 male patients and 50 female patients. There was no significant difference in demographic and clinical characteristics, such as age, sex, height and weight, between the two groups (*p* > 0.05), indicating that the data of the two groups were comparable (see Table 1).

**Table 1:** Baseline characteristics (*n* = 80, mean ± SD)

Features	Control group, *n* = 40	Test group, *n* = 40	*P* value
Age (yrs), Mean (SD)	48.03 ± 15.27	53.20 ± 13.97	0.118
Sex (M/F), N	14/26	16/24	0.644
Height (m), Mean (SD)	165.23 ± 7.73	165.28 ± 8.74	0.978
Body weight (kg), Mean (SD)	67.33 ± 10.59	65.54 ± 9.78	0.435
BMI (kg/m^2^), Mean (SD)	24.61 ± 3.06	23.95 ± 2.72	0.311
Affected side (right/left),N	21/19	21/19	1.000
Duration of pain (months), Median [IQR]	12 (7.25-24.00)	9 (5.25–17.25)	0.315

Note: SD: standard deviation; BMI: body mass index; IQR: interquartile range; according to the normality of the distribution, continuous variables are expressed as the mean ± SD or median [IQR]. Sex is a categorical variable and is represented by N. Experimental group: pain point combined with TrPs treatment. Control group: pain point treatment

### Main effect analysis for the overall NRS score, the NRS score of heel pain at the first step, and the NRS score of heel pain during daily activities over the treatment period for the two groups of patients

The overall NRS score, heel pain NRS score at the first step, and heel pain NRS score during daily activities of the two groups of patients all demonstrated a normal distribution and homogeneity of variance (*p* > 0.05). After Mauchly’s spherical hypothesis test, the covariance matrix of the dependent variable variance was equal, so the results did not need to be corrected by the Greenhouse‒Geisser method.

The results of the overall NRS score, the heel pain NRS score at the first step, and the heel pain NRS score during daily activities of the two groups of patients showed that the time effect was statistically significant (F = 318.328, *p* < 0.001; F = 999.165, *p* < 0.001; F = 1058.978, *p* < 0.001), which reflects that the overall NRS score, heel pain NRS score at the first step, and heel pain NRS score during daily activities of the two groups of patients change over time. There were statistically significant differences in the intergroup effects of the overall NRS score, the NRS score of heel pain at the first step, and the NRS score of heel pain during daily activities between the two groups (F = 20.507, *p* < 0.001). F = 13.438, *p* = 0.001; F = 11.152, *p* < 0.001), which reflects the differences in these scores between the experimental group and the control group. The interaction effects of the overall NRS score, heel pain NRS score at the first step, and heel pain NRS score during daily activities were statistically significant between the two groups (F = 5.452, *p* = 0.006; F = 7.724, *p* = 0.001; F = 8.431, *p* < 0.001), indicating that the influence of time on these scores varied with the treatment method. Therefore, a separate effect analysis should be used to test the time effect and intergroup effect of the two groups of patients (See Table 2).

### Single effect analysis for the total NRS score, the NRS score of heel pain at the first step, and the NRS score of heel pain during daily activities for the two groups over the treatment period

The intragroup comparisons included the overall NRS score, heel pain NRS score at the first step, and heel pain NRS score during daily activities for patients with PF in the experimental group and the control group at 6 weeks and 12 weeks after treatment. The results showed that compared with the baseline values, the overall NRS score, heel pain NRS score during daily activities, and heel pain NRS score at the first step of the two groups decreased at 6 weeks and 12 weeks after treatment (*p* < 0.001).

Comparison between groups: The overall NRS score, the NRS score of heel pain at the first step, and the NRS score of heel pain during daily activities at 6 and 12 weeks after treatment in the experimental group were lower than those in the control group (*p* < 0.001) (See Table 2).

**Table 2:** NRS scores during treatment in both groups (n = 80, mean ± SD)

Index	**Peer group**	Pretreatment baseline	Posttreatment		F, P (time)	F, P (between groups)	F, P (interaction)
			6 week	12 week			
Overall NRS score	Control group (n = 40)	7.13 ± 0.69	3.65 ± 0.86^*^	4.18 ± 0.78^*#^			
	Test group (n = 40)	7.08 ± 0.80	2.95 ± 0.78^*&^	3.3 ± 0.91^*#&^	F = 318.328,	F = 20.507,	F = 5.452,
	t	0.301	3.798	4.611	P < 0.001	P < 0.001	P = 0.006
	P	0.764	< 0.001	< 0.001			
Heel pain at first step	Control group (n = 40)	7.30 ± 0.76	3.73 ± 0.85 ^*^	3.93 ± 0.83 ^*△^			
	Test group (n = 40)	7.28 ± 0.82	3.03 ± 0.73^*&^	3.13 ± 0.79^*#△^	F = 999.165,	F = 13.438,	F = 7.724,
	t	0.142	3.952	4.418	P < 0.001	P = 0.001	P = 0.001
	P	0.887	< 0.001	< 0.001			
Heel pain with daily activities	Control group (n = 40)	7.20 ± 0.79	3.48 ± 0.78 ^*^	3.98 ± 0.77 ^*#^			
	Test group (n = 40)	7.23 ± 0.66	2.88 ± 0.69^*&^	3.25 ± 0.87^*#&^	F = 1058.978,	F = 11.152,	F = 8.431,
	t	-0.154	3.642	3.953	P < 0.001	P < 0.001	P < 0.001
	P	0.878	< 0.001	< 0.001			

* indicates *p* < 0.001 compared to pretreatment, # indicates *p* < 0.05 compared to 6 weeks posttreatment; ^△^indicates *p*>0.05 compared to 6 weeks posttreatment; & indicates *p* < 0.001 compared to the control group

### Comparison of RMS before and after treatment

The percentage of excellent RMSs in the experimental group was significantly increased at 6 and 12 weeks after treatment relative to baseline, while the percentage of excellent grades in the control group was not significantly different from that before the operation. There were significant differences in the RMSs between the two groups at 6 and 12 weeks after treatment (*p* = 0.024, *p* = 0.025), indicating that the experimental group had better results than the control group posttreatment (See Table 3).

**Table 3:** Comparison of Roles–Maudsley scores during treatment between the two groups (*n* = 80)

		Control group (*n* = 40)	Test group (*n* = 40)	χ2	*P*
Pretreatment baseline	difference	85% (*n* = 34)	80% (*n* = 32)	0.346	0.770
	Fair	15% (*n* = 6)	20% (*n* = 8)		
	Good	0 (*n* = 0)	0 (*n* = 0)		
	Excellent	0 (*n* = 0)	0 (*n* = 0)		
6 weeks after treatment	difference	5% (*n* = 2)	0 (*n* = 0)	8.140	0.024
	Fair	50% (*n* = 20)	30% (*n* = 12)		
	Good	45% (*n* = 18)	62.5% (*n* = 25)		
	Excellent	0 (*n* = 0)	7.5% (*n* = 3)		
12 weeks after treatment	difference	10% (*n* = 4)	0 (*n* = 0)	8.498	0.025
	Fair	70% (*n* = 28)	60% (*n* = 24)		
	Good	20% (*n* = 8)	32.5% (*n* = 13)		
	Excellent	0 (*n* = 0)	7.5% (*n* = 3)		

### Main effect analysis of heel temperatures (T1, T2, T3, T4, T5) in the two groups of patients

The heel temperatures (T1, T2, T3, T4, T5) of the two groups of patients demonstrated a normal distribution and homogeneity of variance (*p* > 0.05). After Mauchly’s spherical hypothesis test, the variance covariance matrix of the dependent variables was not equal, so the results needed to be corrected by the Greenhouse‒Geisser method.

The results of the analysis of heel temperatures (T1, T2, T3, T4, T5) in the two groups showed that the time effect was statistically significant (F = 928.892, *p* < 0.001; F = 623.046, *p* < 0.001; F = 943.214, *p* < 0.001; F = 332.332, *p* < 0.001; F = 4470.958, *p* < 0.001), reflecting that the heel temperatures of the two groups of patients changed with time during treatment. There were significant effects of group in the heel temperatures (F = 24.761, *p* < 0.001; F = 13.064, *p* = 0.001; F = 14.304, *p* < 0.001; F = 16.280, *p* < 0.001; F = 21.664, *p* < 0.001), which reflected the difference in heel temperatures between the experimental group and the control group. Heel temperature (T1, T2, T3, T4, T5) also demonstrated significant time-group interaction effects (F = 158.98, *p* < 0.001; F = 69.790, *p* < 0.001; F = 106.332, *p* < 0.001; F = 345.050, *p* < 0.001), indicating that the influence of time on the heel temperature (T1, T2, T3, T4, T5) varied with the treatment method. Therefore, individual effect analyses should be used to test the time effect and intergroup effect for heel temperature (See Table 4).

### Independent effect analysis of the heel temperature (T1, T2, T3, T4, T5) before and after treatment in the two groups of patients

Within-group comparison: The changes in heel temperature (T1, T2, T3, T4, T5) in patients with PF in the experimental group and the control group at 6 weeks and 12 weeks after treatment were compared. The results showed that the plantar temperature (T1, T2, T3, T4, T5) decreased at 6 weeks and 12 weeks after treatment in both groups (*p* < 0.05).

Between-group comparisons: The heel temperatures (T1, T2, T3, T4, T5) of the experimental group at 6 and 12 weeks after treatment were lower than those of the control group (*p* < 0.05) (See Table 4).

**Table 4:** Comparison of the heel temperatures between the two groups (n = 80, mean ± SD)

Index	**Peer group**	Pretreatment baseline	Posttreatment		F,P(time)	F,P (between groups)	F,P **(interaction)**
			6 week	12 week			
T1(℃)	Control group (n = 40)	28.37 ± 0.88	27.9 ± 0.89^*^	28.08 ± 0.89^*#^	F = 928.892, P < 0.001	F = 24.761, P < 0.001	F = 158.098, P < 0.001
	Test group (n = 40)	28.35 ± 0.97	27.33 ± 1.02^*&^	27.44 ± 0.99^*#&^			
	t	0.101	2.658	3.037			
	P	0.920	0.010	0.003			
T2(℃)	Control group (n = 40)	27.99 ± 0.91	27.31 ± 0.91^*^	27.58 ± 0.91^*#^	F = 623.046, P < 0.001	F = 13.064, P = 0.001	F = 69.790, P < 0.001
	Test group (n = 40)	28.02 ± 0.87	26.79 ± 0.81^*&^	27.01 ± 0.81^*#&^			
	t	-0.182	2.723	2.960			
	P	0.856	0.008	0.004			
T3(℃)	Control group (n = 40)	27.92 ± 0.88	27.12 ± 0.86^*^	27.33 ± 0.88^*#^	F = 943.214, P < 0.001	F = 14.304, P < 0.001	F = 80.134, P < 0.001
	Test group (n = 40)	27.96 ± 0.98	26.6 ± 0.96^*&^	26.78 ± 0.90^*#&^			
	t	-0.207	2.533	2.776			
	P	0.836	0.013	0.007			
T4(℃)	Control group (n = 40)	28.03 ± 0.85	27.75 ± 0.79^*^	27.86 ± 0.83^*#^	F = 332.332, P < 0.001	F = 16.280, P < 0.001	F = 106.332, P < 0.001
	Test group (n = 40)	28.08 ± 0.93	27.24 ± 0.87^*&^	27.33 ± 0.90^*#&^			
	t	-0.255	2.769	2.747			
	P	0.800	0.007	0.007			
T5(℃)	Control group (n = 40)	28.55 ± 0.88	27.84 ± 0.84^*^	28.06 ± 0.87^*#^	F = 4470.958, P < 0.001	F = 21.664, P < 0.001	F = 345.050, P < 0.001
	Test group (n = 40)	28.59 ± 0.91	27.31 ± 0.88^*&^	27.33 ± 0.90^*#&^			
	t	-0.208	2.777	3.007			
	P	0.836	0.007	0.007			

* indicates p < 0.001 compared to pretreatment, # indicates p < 0.05 compared to 6 weeks posttreatment; & indicates p < 0.05 compared to the control group

### Comparison of the average and maximum static plantar pressure before and after treatment in the two groups of patients

After 6 weeks of treatment, the average pressure and maximum pressure of the static plantar forefoot of the affected side in the experimental group were significantly smaller than those before treatment, similar to the trend in the control group. After treatment, there was no significant difference in the average pressure and maximum pressure of the affected static plantar forefoot between the two groups before and after treatment (*p* = 0.669, *p* = 0.365). After treatment, the average pressure and maximum pressure of the static plantar hindfoot of the affected side in the experimental group were significantly higher than those before treatment, and the control group showed a similar increasing trend. There was no significant difference in the average pressure or maximum pressure of the affected foot between the two groups before and after treatment (*p* = 0.490, *p* = 0.257). (See Table 5)

### Comparison of the dynamic plantar medial and lateral loads between the two groups before and after treatment

At 6 weeks after treatment, the dynamic medial plantar load of the affected side in the experimental group was significantly reduced compared with that before treatment, and the control group showed the same decreasing trend. There was no significant difference in the dynamic medial plantar load between the two groups before and after treatment (*p* = 0.995). After treatment, the dynamic lateral plantar load of the affected side increased significantly in the experimental group, and the control group showed the same increasing trend. There was no significant difference in the dynamic plantar lateral load between the two groups before and after treatment (*p* = 0.310) (See Table 5).

**Table 5:** Plantar pressure in both groups (*n* = 80, mean ± SD)

Index	**Peer group**	Pretreatment baseline	Posttreatment
			6 week
Forefoot-Mean Static plantar Pressure (gr/cm^2^)	Control group (n = 40)	307.95 ± 58.06	285.18 ± 58.87
	Test group (n = 40)	311.23 ± 59.73	279.80 ± 53.18
	t	-0.249	-0.429
	P	0.804	0.669
hindfoot-Mean Static plantar Pressure (gr/cm^2^)	Control group (n = 40)	344.43 ± 51.53	372.93 ± 51.52
	Test group (n = 40)	347.50 ± 52.95	380.63 ± 47.80
	t	-0.263	-0.693
	P	0.793	0.490
Forefoot- Maximum Static plantar Pressure (gr/cm^2^)	Control group (n = 40)	594.85 ± 69.79	537.15 ± 86.77
	Test group (n = 40)	597.73 ± 70.09	520.05 ± 81.07
	t	-0.184	0.911
	P	0.855	0.365
Hindfoot- Maximum Static plantar Pressure (gr/cm^2^)	Control group (n = 40)	605.73 ± 69.61	637.43 ± 82.00
	Test group (n = 40)	608.8 ± 70.32	657.85 ± 77.94
	t	-0.197	-1.142
	P	0.845	0.257
Dynamic medial load(%)	Control group (n = 40)	17.12 ± 4.69	16.08 ± 4.43
	Test group (n = 40)	17.33 ± 5.08	15.37 ± 4.40
	t	-0.191	-0.006
	P	0.849	0.995
Dynamic lateral load(%)	Control group (n = 40)	13.77 ± 3.73	15.08 ± 3.82
	Test group (n = 40)	13.78 ± 4.58	15.97 ± 3.99
	t	0.719	-1.022
	P	0.474	0.310

## Discussion

TrPs are considered excessive irritability points in skeletal muscle bandages. According to their relationship with the patient’s symptoms, they may cause symptoms such as pain, muscle weakness, and reduced range of motion (ROM) [8]. Recent studies have shown that PF may be caused by TrPs in the myofascial membrane near the pain area, resulting in biomechanical imbalance of the entire limb and pelvis [13, 18]. The TrP inactivation may help relieve muscle tension and spasm, improve local circulation, and thus interrupt the vicious cycle of pain associated with TrPs [18]. Eliminating the active TrPs and hidden TrPs in the gastrocnemius muscle and soleus muscle can also effectively alleviate muscle fatigue and prevent the persistence of overload in the muscle. Therefore, myofascial injury in the lower limb area may affect the mechanical load of the foot and may play a role in the pathogenesis of PF. It has been confirmed in the literature that treating TrPs can effectively improve the pain in PF [19]. At present, the trigger point treatment for PF mainly includes dry needle therapy, injection drug therapy (anesthetics, steroids, botulinum toxin A) and ESWT [12]. As an alternative treatment, steroid injection has been shown to lead to plantar fascia rupture, fat pad atrophy, and even lateral plantar nerve injury [20, 21]. In the past decade, ESWT has been increasingly used worldwide and identified as a relatively safe treatment. Several RCTs have studied PF treatment with extracorporeal shock waves at the medial heel pain point, confirming the effectiveness and safety of ESWT [22]. At present, there are few studies on ESWT combined with TrPs in the treatment of PF.

Temperature is an important parameter reflecting the physiological and pathological state of the human body. Different factors affect the epidermal temperature of the limbs and trunk. The epidermal temperature of the hands and feet is mainly dominated by vasoconstriction and dilatation [23]. Chen et al. [24] found that 3 to 24 months of chronic PF will result in plantar fascia vascularization in the patients, causing changes in blood flow around the area; in addition, the formation of new blood vessels around the plantar fascia often manifests as an increase in the skin temperature of the heel [25]. Danielson et al. [26] found that the level of substance P (SP) in human serum around newly formed vessels was significantly increased in the patellar tendon of patients with patellar tendinitis, so it was speculated that SP was related to neovascularization. Carlsson et al. [27] confirmed, through a study of rat models, that injections of SP into diseased tissue in tendinitis would cause an increase in neovascularization in the tissue. These studies have shown that local temperature may indirectly reflect the level of pain-causing substances in the inflammatory area. Therefore, this study used plantar temperature as an indirect indicator for efficacy evaluation. ESWT can inhibit the excitability of nerve terminal cells and reduce the release of SP [28]. The expression of SP is inhibited, blocking its effect on neovascularization, manifesting as decreased blood flow in the heel and decreased heel temperature. The results of this study showed that the plantar temperature of the experimental group and the control group decreased at 6 and 12 weeks after treatment compared with that before treatment; the decrease in the experimental group was more significant, and the pain improvement was more obvious. However, the plantar temperature at 12 weeks after treatment was slightly higher than that at 6 weeks after treatment. At present, it is believed that the local temperature increase in the heel in patients with PF may be repeated after ESWT. The reasons for the recurrence may be as follows: (1) The treatment time with extracorporeal shock waves is relatively short and cannot completely eliminate the factors inducing PF. The plantar fascia is still accompanied by vascular proliferation. When the treatment is over, it will cause the local temperature of the heel to rise. (2) After ESWT, the degree of pain in patients with PF is reduced, and the amount of activity tends to increase rapidly, resulting in excessive plantar weight-bearing and the reemergence of SP in local tissues. At present, it is not clear whether the local temperature of the heel of patients with PF can be used as an objective index to evaluate the degree of pain. Therefore, it is necessary to further explore the relationship between the two to provide a basis for the evaluation of PF by infrared thermal imaging.

Although gait analysis is considered an objective tool for assessing the progression of PF and the efficacy of treatment options [29], few studies have reported the effect of extracorporeal shock waves combined with TrPs on gait in the treatment of PF [29]. After treatment, the patient increases the load on the painful heel, indicating that the significant improvement in the heel landing stage and plantar pain will cause patients with PF to change the original gait pattern, causing the heel load to decrease and the forefoot load to increase. One possible explanation for this finding is inflammation in the calcaneus region in patients with PF, which may increase the thickness of the plantar fascia, resulting in a decrease in the ability of the tissue to support the mechanical load in the heel region. The reduction of this ability to support load in the plantar fascia may lead to the use of analgesic measures to reduce the subsequent plantar load, resulting in an increase in the plantar load on the forefoot. The results are in line with those of Sullivan [17], and other studies have shown that patients with PF often experience a decrease in the maximum pressure and peak pressure in the heel area. The lower the above value is, the higher the pain degree in patients with PF. This study shows that ESWT will change the local load of the affected foot, and the effect of combining heel pain point with trigger point ESWT is more significant than the traditional simple heel pain point ESWT on the plantar load change. These findings may be related to the recovery of the correct sliding of the fascia system, thereby addressing biomechanical damage. Tension in the proximal muscles, such as contractures in the calf muscles, is likely to be transmitted to the distal muscles through the fascia chain. By treating the contracture of the proximal muscle tissue, the sliding of the entire ‘calf fascia-Achilles tendon-plantar fascia’ structure can be restored, which explains the improvement in foot pressure. However, further studies are needed to determine the relationship between gait improvement, plantar fascia healing and PF-related symptoms after ESWT. In conclusion, heel pain point combined with trigger point ESWT not only reduces the degree of pain in patients with PF but also improves gait parameters, which is helpful for restoring more normal gait patterns in patients with PF.

### Advantages and limitations

The main advantages of this study are as follows: 1. This study may provide a new idea for the treatment of patients with PF: conventional extracorporeal shock waves combined with trigger point therapy provides a new direction for the treatment of PF; and the heel temperature measured by infrared thermal imaging and the plantar data measured by plantar pressure plates provide objective measurement indicators for evaluating the efficacy of PF treatment. The results of this study confirm that ESWT of heel pain points combined with triceps TrPs is a safe and effective conservative treatment for PF.

However, this study also contains several deficiencies: (1) Due to the length of the treatment cycle, we only conducted follow-up after 12 weeks; the long-term clinical effect is not clear, and further follow-up is needed. (2) This study only designated a single treatment parameter but reviewed many published randomized controlled trials, and the results were different [30]. Our results are only applicable to the treatment variables used in this study. Different treatment parameters of ESWT may have different effects on the treatment results. For PF, the optimal dose, parameters and course of treatment of ESWT need to be further explored in the future.

## Conclusion

For refractory PF, ESWT of the heel combined with the gastrocnemius and soleus TrPs can more effectively improve the pain, function and quality of life of patients than simple heel ESWT. In addition, ESWT of the heel combined with the triceps trigger point of the calf can effectively reduce the skin temperature of the heel on the symptomatic side, indicating that the heel temperature measured by infrared thermal imaging may be used as an independent indicator to evaluate the therapeutic effect of patients with chronic PF. Although extracorporeal shock wave combined with TrPs treatment can cause changes in the gait structure of patients, plantar pressure is still difficult to use as an independent tool to evaluate the therapeutic effect of PF.

### Electronic supplementary material

Below is the link to the electronic supplementary material.

Supplementary Material 1

Supplementary Material 2

## Data Availability

The datasets generated and analyzed during the current study are available from the corresponding author upon reasonable request.

## Abbreviations

PF: Plantar fasciitis
ESWT: Extracorporeal shock wave therapy
TrPs: Trigger points
NRS: Numerical Rating Scale
RMS: Roles–Maudsley Score
RCT: Randomized controlled trial
BMI: Body mass index
IQR: Interquartile range
ROM: Range of motion
SP: Substance P
