# Correction: Evaluation of the efficacy of trigger points combined with extracorporeal shock waves in the treatment of plantar fasciitis: heel temperature and plantar pressure

**DOI:** 10.1186/s12891-024-07643-3

**Published:** 2024-07-15

**Authors:** Bo Wang, Xiao-Lei Wang, Yan-Tao Ma, Wei Wu, Yong-Jun Zheng

**Affiliations:** 1 Department of Pain Management, Huadong Hospital Affiliated to Fudan University, 221 West Yan’an RD, Shanghai, China; 2 Department of Elite Sport, School of Athletic Performance, Shanghai University of Sport, 188 Hengren RD, Shanghai, China

**Correction** **:** **BMC Musculoskelet Disord 25, 191 (2024)**

**https://doi.org/10.1186/s12891-024-07296-2**

Following publication of the original article [1], the authors mistakenly used incorrect symbol "T" instead of the correct symbol "t" under Peer group in Tables 2, 4, and 5. The corrected tables are given below.

**Table 2:** NRS scores during treatment in both groups (*n* = 80, mean ± SD)

**Index**	**Peer group**	**Pretreatment baseline**	**Posttreatment**		**F, P (time)**	**F, P (between groups)**	**F, P (interaction)**
			**6 week**	**12 week**			
Overall NRS score	Control group (*n* = 40)	7.13 ± 0.69	3.65 ± 0.86^*^	4.18 ± 0.78^*#^	F = 318.328, *P* < 0.001	F = 20.507, *P* < 0.001	F = 5.452, *P* = 0.006
	Test group (*n* = 40)	7.08 ± 0.80	2.95 ± 0.78^*&^	3.3 ± 0.91^*#&^			
	t	0.301	3.798	4.611			
	P	0.764	< 0.001	< 0.001			
Heel pain at first step	Control group (*n* = 40)	7.30 ± 0.76	3.73 ± 0.85 ^*^	3.93 ± 0.83 ^*△^	F = 999.165, *P* < 0.001	F = 13.438, *P* = 0.001	F = 7.724, *P* = 0.001
	Test group (*n* = 40)	7.28 ± 0.82	3.03 ± 0.73^*&^	3.13 ± 0.79^*#△^			
	t	0.142	3.952	4.418			
	P	0.887	< 0.001	< 0.001			
Heel pain with daily activities	Control group (*n* = 40)	7.20 ± 0.79	3.48 ± 0.78 ^*^	3.98 ± 0.77 ^*#^	F = 1058.978, *P* < 0.001	F = 11.152, *P* < 0.001	F = 8.431, *P* < 0.001
	Test group (*n* = 40)	7.23 ± 0.66	2.88 ± 0.69^*&^	3.25 ± 0.87^*#&^			
	t	-0.154	3.642	3.953			
	P	0.878	< 0.001	< 0.001			

* indicates *p* < 0.001 compared to pretreatment, # indicates *p* < 0.05 compared to 6 weeks posttreatment; ^△^indicates *p* > 0.05 compared to 6 weeks posttreatment; & indicates *p* < 0.001 compared to the control group

**Table 4:** Comparison of the heel temperatures between the two groups (*n* = 80, mean ± SD)

**Index**	**Peer group**	**Pretreatment baseline**	**Posttreatment**		**F,P (time)**	**F,P** **(between groups)**	**F,P (interaction)**
			**6 week**	**12 week**			
T1(℃)	Control group (*n* = 40)	28.37 ± 0.88	27.9 ± 0.89^*^	28.08 ± 0.89^*#^	F = 928.892, *P* < 0.001	F = 24.761, *P* < 0.001	F = 158.098, *P* < 0.001
	Test group (*n* = 40)	28.35 ± 0.97	27.33 ± 1.02^*&^	27.44 ± 0.99^*#&^			
	t	0.101	2.658	3.037			
	P	0.920	0.010	0.003			
T2(℃)	Control group (*n* = 40)	27.99 ± 0.91	27.31 ± 0.91^*^	27.58 ± 0.91^*#^	F = 623.046, *P* < 0.001	F = 13.064, *P* = 0.001	F = 69.790, *P* < 0.001
	Test group (*n* = 40)	28.02 ± 0.87	26.79 ± 0.81^*&^	27.01 ± 0.81^*#&^			
	t	-0.182	2.723	2.960			
	P	0.856	0.008	0.004			
T3(℃)	Control group (*n* = 40)	27.92 ± 0.88	27.12 ± 0.86^*^	27.33 ± 0.88^*#^	F = 943.214, *P* < 0.001	F = 14.304, *P* < 0.001	F = 80.134, *P* < 0.001
	Test group (*n* = 40)	27.96 ± 0.98	26.6 ± 0.96^*&^	26.78 ± 0.90^*#&^			
	t	-0.207	2.533	2.776			
	P	0.836	0.013	0.007			
T4(℃)	Control group (*n* = 40)	28.03 ± 0.85	27.75 ± 0.79^*^	27.86 ± 0.83^*#^	F = 332.332, *P* < 0.001	F = 16.280, *P* < 0.001	F = 106.332, *P* < 0.001
	Test group (*n* = 40)	28.08 ± 0.93	27.24 ± 0.87^*&^	27.33 ± 0.90^*#&^			
	t	-0.255	2.769	2.747			
	P	0.800	0.007	0.007			
T5(℃)	Control group (*n* = 40)	28.55 ± 0.88	27.84 ± 0.84^*^	28.06 ± 0.87^*#^	F = 4470.958, *P* < 0.001	F = 21.664, *P* < 0.001	F = 345.050, *P* < 0.001
	Test group (*n* = 40)	28.59 ± 0.91	27.31 ± 0.88^*&^	27.33 ± 0.90^*#&^			
	t	-0.208	2.777	3.007			
	P	0.836	0.007	0.007			

* indicates *p* < 0.001 compared to pretreatment, # indicates *p* < 0.05 compared to 6 weeks posttreatment; & indicates *p* < 0.05 compared to the control group

**Table 5:** Plantar pressure in both groups (*n* = 80, mean ± SD)

**Index**	**Peer group**	**Pretreatment baseline**	**Posttreatment**
			**6 week**
Forefoot-Mean Static plantar Pressure (gr/cm^2^)	Control group (*n* = 40)	307.95 ± 58.06	285.18 ± 58.87
	Test group (*n* = 40)	311.23 ± 59.73	279.80 ± 53.18
	t	-0.249	-0.429
	P	0.804	0.669
hindfoot-Mean Static plantar Pressure (gr/cm^2^)	Control group (*n* = 40)	344.43 ± 51.53	372.93 ± 51.52
	Test group (*n* = 40)	347.50 ± 52.95	380.63 ± 47.80
	t	-0.263	-0.693
	P	0.793	0.490
Forefoot- Maximum Static plantar Pressure (gr/cm^2^)	Control group (*n* = 40)	594.85 ± 69.79	537.15 ± 86.77
	Test group (*n* = 40)	597.73 ± 70.09	520.05 ± 81.07
	t	-0.184	0.911
	P	0.855	0.365
Hindfoot- Maximum Static plantar Pressure (gr/cm^2^)	Control group (*n* = 40)	605.73 ± 69.61	637.43 ± 82.00
	Test group (*n* = 40)	608.8 ± 70.32	657.85 ± 77.94
	t	-0.197	-1.142
	P	0.845	0.257
Dynamic medial load(%)	Control group (*n* = 40)	17.12 ± 4.69	16.08 ± 4.43
	Test group (*n* = 40)	17.33 ± 5.08	15.37 ± 4.40
	t	-0.191	-0.006
	P	0.849	0.995
Dynamic lateral load(%)	Control group (*n* = 40)	13.77 ± 3.73	15.08 ± 3.82
	Test group (*n* = 40)	13.78 ± 4.58	15.97 ± 3.99
	t	0.719	-1.022
	P	0.474	0.310

The original article [1] has been corrected.
