# Supplementary material for: Evaluation of the efficacy of trigger points combined with extracorporeal shock waves in the treatment of plantar fasciitis: heel temperature and plantar pressure
Source: BMC Musculoskelet Disord. 2024 Mar 2;25:191. doi: 10.1186/s12891-024-07296-2 (PMC10908045; doi:10.1186/s12891-024-07296-2)
Supplement: Supplementary file 1 — Supplementary Material 1 [file 12891_2024_7296_MOESM1_ESM.doc]

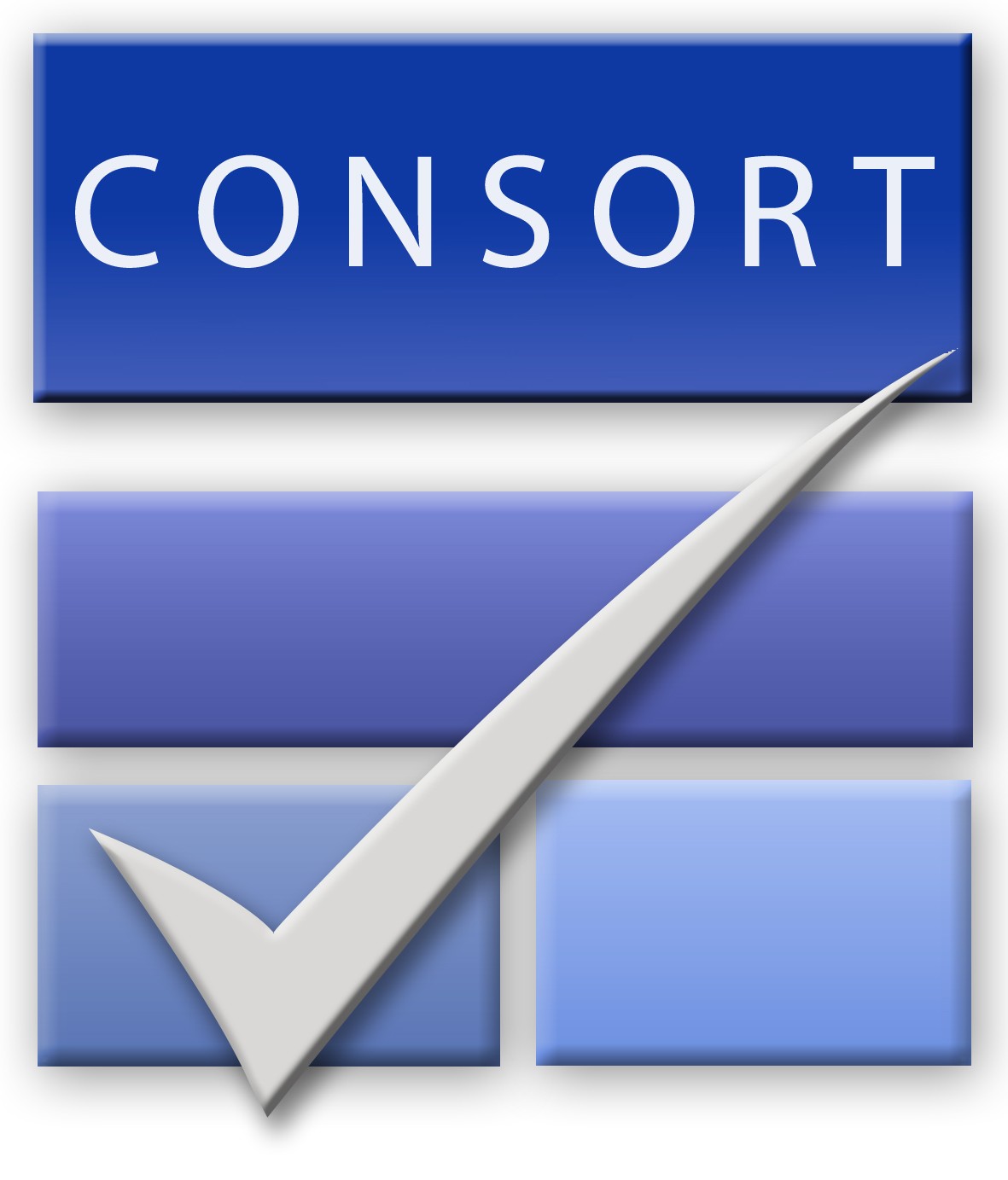
CONSORT 2010 checklist of information to include when reporting a randomised trial*

| Section/Topic | Item No | Checklist item | Reported on page No |
| --- | --- | --- | --- |
| Title and abstract | | | |
|  | 1a | Identification as a randomised trial in the title | None |
|  | 1b | Structured summary of trial design, methods, results, and conclusions (for specific guidance see CONSORT for abstracts) | Page 3, lines 41-48, Page 4, lines49-70，and Page 5，lines 71-77 |
| Introduction | | | |
| Background and objectives | 2a | Scientific background and explanation of rationale | Page 5, lines 84-92, Page 6 lines93-114, and Page7, lines115-123 |
|  | 2b | Specific objectives or hypotheses | Page7, lines123-128 |
| Methods | | | |
| Trial design | 3a | Description of trial design (such as parallel, factorial) including allocation ratio | Page 7, lines131-132，and Page 10, lines179-182 |
|  | 3b | Important changes to methods after trial commencement (such as eligibility criteria), with reasons | None |
| Participants | 4a | Eligibility criteria for participants | Page 9, lines160-175 |
|  | 4b | Settings and locations where the data were collected | Page 8, lines152-154 |
| Interventions | 5 | The interventions for each group with sufficient details to allow replication, including how and when they were actually administered | Page 13, lines251-252, Page 14, lines253-274, Page 15, lines275-276 |
| Outcomes | 6a | Completely defined pre-specified primary and secondary outcome measures, including how and when they were assessed | Page 11, lines200-212, Page 12, lines213-234，and Page 13, lines235-247 |
|  | 6b | Any changes to trial outcomes after the trial commenced, with reasons | None |
| Sample size | 7a | How sample size was determined | Page 8 lines142-150 |
|  | 7b | When applicable, explanation of any interim analyses and stopping guidelines | None |
| Randomisation: |  |  |  |
| Sequence generation | 8a | Method used to generate the random allocation sequence | Page 10, lines179-180 |
|  | 8b | Type of randomisation; details of any restriction (such as blocking and block size) | None |
| Allocation concealment mechanism | 9 | Mechanism used to implement the random allocation sequence (such as sequentially numbered containers), describing any steps taken to conceal the sequence until interventions were assigned | Page10, lines179-187 |
| Implementation | 10 | Who generated the random allocation sequence, who enrolled participants, and who assigned participants to interventions | Page 10, lines181-182, Page 10, lines186-187 |
| Blinding | 11a | If done, who was blinded after assignment to interventions (for example, participants, care providers, those assessing outcomes) and how | Page10, lines184-185 |
|  | 11b | If relevant, description of the similarity of interventions | None |
| Statistical methods | 12a | Statistical methods used to compare groups for primary and secondary outcomes | Page 15, lines278-288 |
|  | 12b | Methods for additional analyses, such as subgroup analyses and adjusted analyses | and Page 15, lines288-296, and Page 16, lines297-301 |
| Results | | | |
| Participant flow (a diagram is strongly recommended) | 13a | For each group, the numbers of participants who were randomly assigned, received intended treatment, and were analysed for the primary outcome | None |
|  | 13b | For each group, losses and exclusions after randomisation, together with reasons | Page16, lines306-312 |
| Recruitment | 14a | Dates defining the periods of recruitment and follow-up | None |
|  | 14b | Why the trial ended or was stopped | None |
| Baseline data | 15 | A table showing baseline demographic and clinical characteristics for each group | Page 17, lines318-324 |
| Numbers analysed | 16 | For each group, number of participants (denominator) included in each analysis and whether the analysis was by original assigned groups | none |
| Outcomes and estimation | 17a | For each primary and secondary outcome, results for each group, and the estimated effect size and its precision (such as 95% confidence interval) | Page20, lines373-375 Page 20, lines387-388，Page 25, lines428，Page 26, lines429-430，Page 29, lines461 |
|  | 17b | For binary outcomes, presentation of both absolute and relative effect sizes is recommended | None |
| Ancillary analyses | 18 | Results of any other analyses performed, including subgroup analyses and adjusted analyses, distinguishing pre-specified from exploratory | None |
| Harms | 19 | All important harms or unintended effects in each group (for specific guidance see CONSORT for harms) | Page10, lines176-177 |
| Discussion | | | |
| Limitations | 20 | Trial limitations, addressing sources of potential bias, imprecision, and, if relevant, multiplicity of analyses | Page 36, lines578-587 |
| Generalisability | 21 | Generalisability (external validity, applicability) of the trial findings | Page 35, lines569-575，and Page 36, lines576-577 |
| Interpretation | 22 | Interpretation consistent with results, balancing benefits and harms, and considering other relevant evidence | Page36, lines581-583 |
| Other information | | |  |
| Registration | 23 | Registration number and name of trial registry | Page 37 lines607-613 |
| Protocol | 24 | Where the full trial protocol can be accessed, if available | Page 37 lines617-618 |
| Funding | 25 | Sources of funding and other support (such as supply of drugs), role of funders | Page 38 lines624-628 |

*We strongly recommend reading this statement in conjunction with the CONSORT 2010 Explanation and Elaboration for important clarifications on all the items. If relevant, we also recommend reading CONSORT extensions for cluster randomised trials, non-inferiority and equivalence trials, non-pharmacological treatments, herbal interventions, and pragmatic trials. Additional extensions are forthcoming: for those and for up to date references relevant to this checklist, see [www.consort-statement.org](http://www.consort-statement.org/).
