# Supplementary material for: Evaluation of the efficacy of trigger points combined with extracorporeal shock waves in the treatment of plantar fasciitis: heel temperature and plantar pressure
Source: BMC Musculoskelet Disord. 2024 Mar 2;25:191. doi: 10.1186/s12891-024-07296-2 (PMC10908045; doi:10.1186/s12891-024-07296-2)
Supplement: Supplementary file 2 — Supplementary Material 2 [file 12891_2024_7296_MOESM2_ESM.docx]

### Research protocol

### part 1

### Project summary

**Background:** Plantar fasciitis (PF) is the most common cause of heel pain. Among conservative treatments, extracorporeal shock wave therapy (ESWT) is considered effective for refractory PF. Studies have shown that applying ESWT to the trigger points (TrPs) in the triceps surae may play an important role in pain treatment in patients with PF.Therefore, the purpose of this study was to combine the concept of trigger points and ESWT to explore the effect of this combination on plantar temperature and pressure in patients with PF.

**Methods:** After applying inclusion and exclusion criteria, 86 patients with PF were recruited from the pain clinic of Huadong Hospital, Fudan University and randomly divided into experimental (n = 43) and control groups (n = 43). The experimental group was treated with extracorporeal shock waves to treat the medial heel pain point and the gastrocnemius and soleus TrPs. The control group was only treated with extracorporeal shock waves at the medial heel pain point. The two groups were treated twice with an interval of 1 week. Primary measurements included a numerical rating scale (NRS) score(overall, first step, heel pain during daily activities), and secondary measurements included heel temperature, Roles-Maudsley score (RMS), and plantar pressure. All assessments were performed before treatment (i.e., baseline) and 6 and 12 weeks after treatment.

**Results:** During the trial, 3 patients in the experimental group withdrew from the study, 2 due to interruption of the course of treatment by the COVID-19 epidemic and 1due to personal reasons. In the control group, 3 patients fell and were removed due to swelling of the heel. Therefore, only 80 patients with PF were finally included. After treatment, the two groups showed good results in NRS score(overall, first step, heel pain during daily activities), RMS, and plantar temperature, especially in the experimental group, who show a significantly better effect than the control group.

**Conclusions:**

ESWT of the heel combined with the triceps trigger point of the calf can more effectively improve the pain, function and quality of life of refractory PF than ESWT of the heel alone. In addition, ESWT of the heel combined with the triceps trigger point of the calf can effectively reduce the skin temperature of the heel on the symptomatic side, indicating that the heel temperature as measured by infrared thermal imaging may be used as an independent tool to evaluate the therapeutic effect for patients with chronic PF. Although extracorporeal shock waves combined with TrPs treatment can cause changes in the patients’ gait structure, plantar pressure is still difficult to use as an independent tool to evaluate the therapeutic effect for PF.

### General information

Evaluation of the efficacy of trigger points combined with extracorporeal shock waves in the treatment of plantar fasciitis: heel temperature and plantar pressure.

This study was supported by the Science and Technology Commission of Shanghai Municipality (grant number 22Y11912600),Key Disciplines of Huadong Hospital Affiliated to Fudan University (grant number ZDXK2215),and Key Special Disease Project of Huadong Hospital Affiliated to Fudan University (grant number ZDZB2219).

1. Bo Wang, M.S, Department of Pain Management, Huadong Hospital affiliated to Fudan University, Shanghai, China.

2. Xiao-Lei Wang, M.S, Department of Pain Management, Huadong Hospital affiliated to Fudan University, Shanghai, China.

3. Wei Wu, Ph.D, Department of Elite Sport, School of Athletic Performance, Shanghai University of Sport, Shanghai, China

4. Yong-Jun Zheng, Ph.D, Department of Pain Management, Huadong Hospital affiliated to Fudan University, Shanghai, China.

5. Yan-Tao Ma, M.S, Department of Pain Management, Huadong Hospital affiliated to Fudan University, Shanghai, China.

Conception and design: Bo Wang, Wei Wu, Yong-Jun Zheng; Acquisition of data: Yan-Tao Ma Analysis and interpretation of the data: Xiao-Lei Wang; Drafting of the article: Bo Wang, Xiao-Lei Wang; Critical revision of the article for important intellectual content: Wei Wu. All authors contributed to and approved the final manuscript.

the address and telephone number(s) of the research site(s): Department of Pain Management, Huadong Hospital affiliated to Fudan University,221 West Yan'an RD, Shanghai,China Tel.18116267895

### Rationale & background information

Plantar fasciitis (PF) is a degeneration of the plantar fascia resulting from repetitive microtears that lead to an inflammatory reaction and not a primary inflammatory process as most believe it to be^[1]^. In most cases, PF is a self-limiting disease, but complete remission of symptoms may take up to 1 year^[2]^.

PF can be treated by surgery or conservative treatment^[3]^. Due to the large trauma associated with surgical treatment and the risk of postoperative complications, conservative treatment is the first choice in clinical practice^[4]^. Conservative treatment is effective for approximately 90% of patients^[5]^and includes nonsteroidal anti-inflammatory drugs, plantar corrective insoles, physical therapy, stretching, corticosteroid injections, etc. Extracorporeal shock wave therapy(ESWT) is considered the main means of conservative treatment for PF，providing substantial symptom relief for most patients^[6]^. However, most ESWT schemes commonly used to treat PF only focus on the plantar fascia and do not consider the tension of the entire lower limb,and the curative effect is often of a short duration.

At present, a relationship between trigger points(TrPs) and PF symptoms is acknowledge^[7-9^]. TrPs are sensitive nodules in the muscle or fascia that are painful when palpated, resulting in distal referred pain and autonomic nerve response^[8, 10]^. TrPs are mainly formed by excessive use of skeletal muscle and can be divided into two states: activated or recessive. Activated TrPs cause local area pain and referred pain, while recessive TrPs require mechanical stimulation to cause pain[8, 11]. At present, there are few studies on the use of extracorporeal shock waves combined with TrPs in the treatment of PF. Commonly used evaluation methods, such as the digital scoring method, are subjective and cannot objectively reflect the therapeutic effect^[12, 13]^.

### Some studieshave suggested that PF may be caused by inflammation^[14]^, whose reduction or aggravation can be refelected by a decrease or increase in temperature,respectively.The presence of TrPs induces excessive excitation of the sympathetic nerve, whose main function is to regulate blood vessels, resulting in local skin temperature changes^[15]^. Therefore, when the distribution of blood vessels in the heel changes, the temperature of the heel will change accordingly. Other studies have suggested that PF may be caused by high tension stimulation of the plantar fascia overload, and the presence of TrPs will cause higher tension than that in normal tissue^[12]^.

### Study goals and objectives

In this study, we hypothesized that ESWT combined with TrPs treatment would reduce the heel temperature and cause changes in plantar pressure parameters in patients with PF. Therefore, our aim was to investigate the effect of extracorporeal shock waves combined with TrPs on plantar temperature and pressure in patients with PF.

### Study design

A single-blind trial with 12 weeks of follow-up for parallel groups was carried out from August 2021 to August 2022. The study was conducted according to the principles of the Helsinki Declaration and approved by the Ethics Committee of Huadong Hospital Affiliated with Fudan University: No. 2021K109. This randomized controlled trial (RCT) has been registered at the Primary Registry of International Clinical Trial Registry Platform World Health Organization “Chinese Clinical Trial Registry” [ChiCTR2100054439]. Before participating in the project, the purpose of the study was explained to all patients, and written informed consent was obtained from all patients.

**Sample size**

Due to the lack of previous studies, this study estimated the sample size through a preexperiment. This study is an RCT of a parallel design, with a numerical rating scale(NRS) as the main observation index, which is a continuous variable. The sample size was calculated by PASS 15 software. The test level parameter was set to α = 0.05 (bilateral), and the test efficiency parameter was set to 1-β = 80%. The minimum sample size required for the experimental group and the control group was calculated as N1 = N2 = 23 cases. The exit rate was set to 20%, so at least 29 people should be included in each group.

**Participants**

A total of 118 participants were assessed for eligibility. Eighty-six patients with PF were recruited from the Department of Pain Management, Huadong Hospital Affiliated with Fudan University. All of the patients refused to undergo any surgery and signed informed consent forms. The CONSORT flow diagram for an RCT to evaluate the efficacy of extracorporeal shock waves combined with TrPs in patients with PF is illustrated in Fig. 1.

All patients voluntarily participated in the experiment. The inclusion criteria were as follows: 1.18 years of age or older; 2. heel painalsting≥ 3 months; and 3. Pain in the first step after waking up in the morning or obvious pain in the medial calcaneal tubercle and the starting point of the plantar fascia at 2-3cm afterstanding for an extended period as well as, B-mode ultrasonography showing plantar aponeurosis thickening greater than 4 cm and a low echogenic area^[16]^and an NRS score≥ 5 points; 4. Ineffective previous conservative treatment (nonsteroidal anti-inflammatory drugs and/or other analgesics, exercise programs, insoles); and 5. signed informed consent. The exclusion criteria were as follows: 1. Previous history of ankle and foot fracture or surgery, foot and ankle infection, or history of lower limb tumor; 2. lower extremity neurological dysfunction; 3. rheumatic diseases and metabolic diseases; and 4. local injection of steroids or surgical treatment within three months.

**Randomization and blinding**

Using a computer-generated random allocation sequence,86 patients with PF were randomized and assigned into two equal groups by an independent researcher: an experimental group and a control group^[17]^. An independent researcher who was not involved in the recruitment, assessment, or intervention process conducted the randomization and was blinded to the group allocation. Participants were also instructed not to reveal their group assignment. Sequentially numbered sealed envelopes were used for allocation. The envelopes were opened only by the researcher responsible for applying the treatment programs.In group A,the experimental group, the treatment target was selected as the ipsilateral TrPs (gastrocnemius and soleus TrPs) and heel medial area pain points, while in group B,the control group, the treatment target was selected as the pain point in the medial area of the affected heel. Both groups were treated with a Swiss Storzradial shock wave device. The energy flux density is usually considered the main variable reflecting the physical and biological effects to maintain the consistency of treatment. A total of 3,000 shockswith a 15 mm treatment probe were selected. 8 Hz), and the treatment intensity was 1.4 bar.

Study execute time：from August 2021 to August 2022.

### Methodology

**Experimental group**

According to the internationally recognized three principles of trigger point positioning theory, the following were used for location: 1. palpable skeletal muscle on tight band nodules; 2. obvious tenderness points on the tight bandage; and 3. Reappearance of the patients’ symptoms of pain during palpation. If a point satisfies the above three conditions, it is considered an active trigger point. If it satisfies both 1 and 2 but does not satisfy 3, it is considered an implicit trigger point^[8]^. The patient's gastrocnemius muscle, soleus muscle activity and hidden TrPs were identified and marked with a medical sterile surgical marker pen. To evaluate the trigger point position at the next session, the patient was asked not to clean the marker mark. Diagnostic criteria were applied by physical therapists with five years of experience in myofascial pain.The Swiss Storz-MP200 radial ESWT device was used once a week for a total of 2 treatments. The TrPs of the triceps surae and the pain points in the medial area of the heel were taken as the treatment points. Each patient was placed in a prone position, and the patient’s hands were placed on the side of the body to fully expose the affected leg and heel. The coupling agent was applied at the specified position, the leg was subjected to vertical impact, the heel was subjected to lateral impact, and 300 impacts were applied at each trigger point.

**Control group**

The subjects were treated with the Swiss Storz-MP200 radial ESWT at the medial heel pain point identified after palpation; the course of treatment, frequency and dose were the same as those of the experimental group.

#### Outcome measurement

Patients were measured and evaluated by a single person to reduce errors. All patients were assessed with the digital NRS, the Roles-Maudsley score(RMS), and infrared thermal imaging to measure heel temperature and plantar pressure before treatment (i.e., baseline) and 6 weeks after treatment and with the NRS, the RMS and infrared thermal imaging to measure heel temperature at 12 weeks after treatment.

### Safety considerations

During the trial, 3 patients in the experimental group withdrew from the study, 2 due to interruption of the course of treatment by the COVID-19 epidemic and 1due to personal reasons. In the control group, 3 patients fell and were removed due to swelling of the heel. Therefore, only 80 patients with PF were finally included.

### Follow-up

 The study was followed up for 12 weeks and all subjects were informed by telephone to Huadong Hospital Affiliated to Fudan University for examination.

### Data management and statistical analysis

Statistical Package for Social Science 23.0 was used for statistical analysis. Continuous variables that conformed to a normal distribution are expressed as the mean and standard deviation. The Kolmogorov-Smirnov(K-S) test was used to test whether the data obeyed a normal distribution. If the data obeyed a normal distribution, the independent t test was used to compare data between groups;otherwise,a nonparametric test was used, and the Mann‒Whitney U test was used for pairwise comparisons. Categorical variables (sex) are expressed as frequencies and composition ratios, and the chi-square test was used to compare groups. Repeated measurement analyses were implemented for efficacy indicators that met a normal distribution and homogeneity of variance. If the Mauchly sphericity test wassuccessful, two-factor analysis of variance was used;otherwise, the Greenhouse‒Geisser method was used for result correction. In the results of repeated measures analysis of variance, if there was no interaction effect between time and treatment factors, the main effect test was directly used to To evaluate the trigger point position evaluate the effect of treatment factors. If there was an interaction effect between time and treatment factors, the individual effect was analyzed; that is, the intragroup effect was analyzed by one-way repeated measurement analysis of variance, and the intergroup effect was analyzed by multivariate analysis of variance. P < 0.05 indicated that the difference was statistically significant, and P < 0.01 indicated a significant difference.

Quality assurance

 This study was monitored by the the Ethics Committee of Huadong Hospital Affiliated to Fudan University

### Expected outcomes of the study

For refractory PF, ESWT of the heel combined with the gastrocnemius and soleus TrPs can more effectively improve the pain, function and quality of life of patients than simple heel ESWT. In addition,ESWT of the heel combined with the triceps trigger point of the calf can effectively reduce the skin temperature of the heel on the symptomatic side, indicating that the heel temperature measured by infrared thermal imaging may be used as an independent indicator to evaluate the therapeutic effect of patients with chronic PF. Although extracorporeal shock wave combined with TrPs treatment can cause changes in the gait structure of patients, plantar pressure is still difficult to use as an independent tool to evaluate the therapeutic effect of PF.

### Dissemination of results and publication policy

Yong-Jun Zheng willtake the lead in publication and will be acknowledged in publications

### Duration of the project

Study execute time：from August 2021 to August 2022.

### Problems anticipated

This study was supported by the Science and Technology Commission of Shanghai Municipality (grant number 22Y11912600)、Key disciplines of Huadong Hospital Affiliated to Fudan University (grant number ZDXK2215)、Key Special Disease Project of Huadong Hospital Affiliated to Fudan University(grant number ZDZB2219), and the project funds are sufficient to meet the experimental needs

### Project management

Conception and design: Bo Wang, Wei Wu, Yong-Jun Zheng; Acquisition of data: Yan-Tao Ma Analysis and interpretation of the data: Xiao-Lei Wang; Drafting of the article: Bo Wang, Xiao-Lei Wang; Critical revision of the article for important intellectual content: Wei Wu. All authors contributed to and approved the final manuscript.

### Ethics

The study was conducted according to the principles of the Helsinki Declaration and approved by the Ethics Committee of Huadong Hospital Affiliated to Fudan University: No. 2021K109. This randomized controlled trial (RCT) has been registered at the Primary Registry of International Clinical Trial Registry Platform World Health Organization “Chinese Clinical Trial Registry” [ChiCTR2100054439].

### Informed consent forms

the Informed consent forms is available in Related files

## References

**References**

[1] Luffy L, Grosel J, Thomas R, et al. Plantar fasciitis: A review of treatments[J]. JAAPA, 2018,31(1):20-24.

[2] Ferreira G F, Sevilla D, Oliveira C N, et al. Comparison of the effect of hyaluronic acid injection versus extracorporeal shockwave therapy on chronic plantar fasciitis: Protocol for a randomized controlled trial[J]. PLoS One, 2021,16(6):e250768.

[3] Trojian T, Tucker A K. Plantar Fasciitis[J]. Am Fam Physician, 2019,99(12):744-750.

[4] Atzmon R, Eilig D, Dubin J, et al. Comparison of Platelet-Rich Plasma Treatment and Partial Plantar Fasciotomy Surgery in Patients with Chronic Plantar Fasciitis: A Randomized, Prospective Study[J]. J Clin Med, 2022,11(23).

[5] Engkananuwat P, Kanlayanaphotporn R, Purepong N. Effectiveness of the Simultaneous Stretching of the Achilles Tendon and Plantar Fascia in Individuals With Plantar Fasciitis[J]. Foot Ankle Int, 2018,39(1):75-82.

[6] Ibrahim M I, Donatelli R A, Schmitz C, et al. Chronic plantar fasciitis treated with two sessions of radial extracorporeal shock wave therapy[J]. Foot Ankle Int, 2010,31(5):391-397.

[7] Ajimsha M S, Binsu D, Chithra S. Effectiveness of myofascial release in the management of plantar heel pain: a randomized controlled trial[J]. Foot (Edinb), 2014,24(2):66-71.

[8] Ortega-Santiago R, Rios-Leon M, Martin-Casas P, et al. Active Muscle Trigger Points Are Associated with Pain and Related Disability in Patients with Plantar Heel Pain: A Case-Control Study[J]. Pain Med, 2020,21(5):1032-1038.

[9] Thummar R C, Rajaseker S, Anumasa R. Association between trigger points in hamstring, posterior leg, foot muscles and plantar fasciopathy: A cross- sectional study[J]. J Bodyw Mov Ther, 2020,24(4):373-378.

[10] Cotchett M P, Landorf K B, Munteanu S E. Effectiveness of dry needling and injections of myofascial trigger points associated with plantar heel pain: a systematic review[J]. J Foot Ankle Res, 2010,3:18.

[11] Renan-Ordine R, Alburquerque-Sendin F, de Souza D P, et al. Effectiveness of myofascial trigger point manual therapy combined with a self-stretching protocol for the management of plantar heel pain: a randomized controlled trial[J]. J Orthop Sports Phys Ther, 2011,41(2):43-50.

[12] Moghtaderi A, Khosrawi S, Dehghan F. Extracorporeal shock wave therapy of gastroc-soleus trigger points in patients with plantar fasciitis: A randomized, placebo-controlled trial[J]. Adv Biomed Res, 2014,3:99.

[13] Tognolo L, Giordani F, Biz C, et al. Myofascial points treatment with focused extracorporeal shock wave therapy (f-ESWT) for plantar fasciitis: an open label randomized clinical trial[J]. Eur J Phys Rehabil Med, 2022,58(1):85-93.

[14] Thompson J V, Saini S S, Reb C W, et al. Diagnosis and management of plantar fasciitis[J]. J Am Osteopath Assoc, 2014,114(12):900-906.

[15] Cao L, Gao Y, Wu K, et al. Sympathetic hyperinnervation in myofascial trigger points[J]. Med Hypotheses, 2020,139:109633.

[16] Rhim H C, Kwon J, Park J, et al. A Systematic Review of Systematic Reviews on the Epidemiology, Evaluation, and Treatment of Plantar Fasciitis[J]. Life (Basel), 2021,11(12).

[17] Ribeiro A P, Joao S M, Dinato R C, et al. Dynamic Patterns of Forces and Loading Rate in Runners with Unilateral Plantar Fasciitis: A Cross-Sectional Study[J]. PLoS One, 2015,10(9):e136971.

[18] Ye L, Mei Q, Li M, et al. A comparative efficacy evaluation of ultrasound-guided pulsed radiofrequency treatment in the gastrocnemius in managing plantar heel pain: a randomized and controlled trial[J]. Pain Med, 2015,16(4):782-790.

[19] Juchli L. Effectiveness of Massage Including Proximal Trigger Point Release for Plantar Fasciitis: a Case Report[J]. Int J Ther Massage Bodywork, 2021,14(2):22-29.

[20] Acevedo J I, Beskin J L. Complications of plantar fascia rupture associated with corticosteroid injection[J]. Foot Ankle Int, 1998,19(2):91-97.

[21] Sellman J R. Plantar fascia rupture associated with corticosteroid injection[J]. Foot Ankle Int, 1994,15(7):376-381.

[22] Gerdesmeyer L, Frey C, Vester J, et al. Radial extracorporeal shock wave therapy is safe and effective in the treatment of chronic recalcitrant plantar fasciitis: results of a confirmatory randomized placebo-controlled multicenter study[J]. Am J Sports Med, 2008,36(11):2100-2109.

[23] Hedrick M R. The plantar aponeurosis[J]. Foot Ankle Int, 1996,17(10):646-649.

[24] Chen H, Ho H M, Ying M, et al. Association between plantar fascia vascularity and morphology and foot dysfunction in individuals with chronic plantar fasciitis[J]. J Orthop Sports Phys Ther, 2013,43(10):727-734.

[25] Lemont H, Ammirati K M, Usen N. Plantar fasciitis: a degenerative process (fasciosis) without inflammation[J]. J Am Podiatr Med Assoc, 2003,93(3):234-237.

[26] Danielson P, Alfredson H, Forsgren S. Distribution of general (PGP 9.5) and sensory (substance P/CGRP) innervations in the human patellar tendon[J]. Knee Surg Sports Traumatol Arthrosc, 2006,14(2):125-132.

[27] Carlsson O, Schizas N, Li J, et al. Substance P injections enhance tissue proliferation and regulate sensory nerve ingrowth in rat tendon repair[J]. Scand J Med Sci Sports, 2011,21(4):562-569.

[28] Notarnicola A, Moretti B. The biological effects of extracorporeal shock wave therapy (eswt) on tendon tissue[J]. Muscles Ligaments Tendons J, 2012,2(1):33-37.

[29] Brachman A, Sobota G, Marszalek W, et al. Plantar pressure distribution and spatiotemporal gait parameters after the radial shock wave therapy in patients with chronic plantar fasciitis[J]. J Biomech, 2020,105:109773.

[30] Sun J, Gao F, Wang Y, et al. Extracorporeal shock wave therapy is effective in treating chronic plantar fasciitis: A meta-analysis of RCTs[J]. Medicine (Baltimore), 2017,96(15):e6621.

### part 2

### Budget

 The research has not been budgeted, and the actual expenses are paid according to actual expenses

### Other support for the project

None

### Collaboration with other scientists or research institutions

None

### Links to other projects

None

### Curriculum Vitae of investigators

None

### Other research activities of the investigators

None

### Financing and insurance

None
